# Determining ICU EEG periodic patterns and why it matters

**DOI:** 10.1007/s00415-023-11835-7

**Published:** 2023-07-01

**Authors:** Philippe Gélisse, William O. Tatum, Arielle Crespel, Pierre Jallon, Peter W. Kaplan

**Affiliations:** 1 Epilepsy Unit, Hôpital Gui de Chauliac, 80 Avenue Fliche, 34295 Montpellier Cedex 05, France; 2 Research Unit (URCMA: Unité de Recherche sur les Comportements et Mouvements Anormaux), INSERM, U661, Montpellier, France; 3 Department of Neurology, Mayo Clinic College of Medicine and Health Sciences, Jacksonville, FL USA; 4 University Medical Center, Ho Chi Minh City, Vietnam; 5 Epilepsy and EEG Unit, Johns Hopkins Bayview Medical Center, Baltimore, MD USA

**Keywords:** Electroencephalogram, Periodic, Rhythmic, Triphasic waves, Generalized periodic discharges, Nonconvulsive status epilepticus

## Abstract

Historically, periodic EEG patterns were described as any pattern with stereotyped paroxysmal complexes occurring at regular intervals, i.e., the period (*T*). *T* is the sum of the duration of the waveform (t1) and, eventually, the duration of the interval between two consecutive waves (t2). The American Clinical Neurophysiology Society introduced the concept of a clearly discernible inter-discharge interval between consecutive waveforms (i.e., t2). As this definition was not applied to what have previously been termed triphasic waves and in some cases of lateralized periodic discharges, we propose reconsideration of terminology that includes historical use of definitions. This will allow the development and usage of the concept for periodic EEG patterns as *any runs of stereotyped paroxysmal waveforms separated by nearly identical intervals and prolonged repetitive complexes on the EEG*. Prolonged expression means EEG is recorded for a sufficient period of time to prove that the pattern is repetitive, thus resulting in a monomorphic/monotonous pattern. More important than the inter-discharge interval (t2), periodic EEG patterns occur at time regular intervals (T). As a result, periodic EEG activity should be considered along a continuum and not the opposite of rhythmic EEG activity where no interval activity exists between consecutive waveforms.

## Introduction

**Fig. 1:**
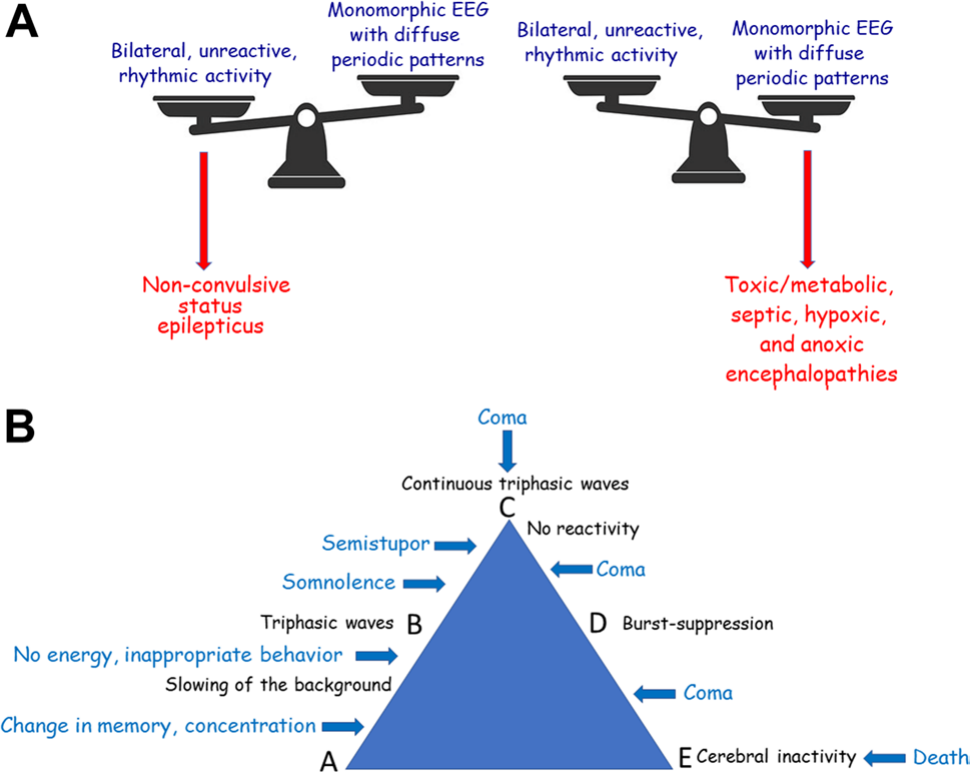
**A** Non-convulsive status epilepticus (NCSE) versus acute encephalopathy. In NCSE, the continuous discharges are unreactive to sensory stimulations. But in metabolic/toxic, septic, hypoxic encephalopathies with triphasic waves, the periodic pattern can be transiently abolished when the patient is fully awake, either spontaneously or induced by auditory or painful stimulation [24]. **B** Clinical and EEG evolution in hepatic encephalopathies. Hepatic encephalopathy was selected as a clinical model to represent a number of entities capable of demonstrating a similar dynamic pattern in the EEG that is able to evolve over time

An EEG report should correspond to a formal and orderly process [1]. In the first part, the recordings must be described (background activity, hyperventilation, intermittent light stimulation, level of alertness, reactivity, abnormal or unusual EEG patterns…) then a conclusion must be given. The importance of determining “periodic” versus “rhythmic” features should always be considered when analyzing an EEG in critically ill patients. Relevance lies with a balance that frequently supports an acute encephalopathy (metabolic/toxic, septic, hypoxic, anoxic) for diffuse periodic patterns and status epilepticus for unreactive bilateral rhythmic patterns within the background activity (Fig. 1A).

**Fig. 2:**
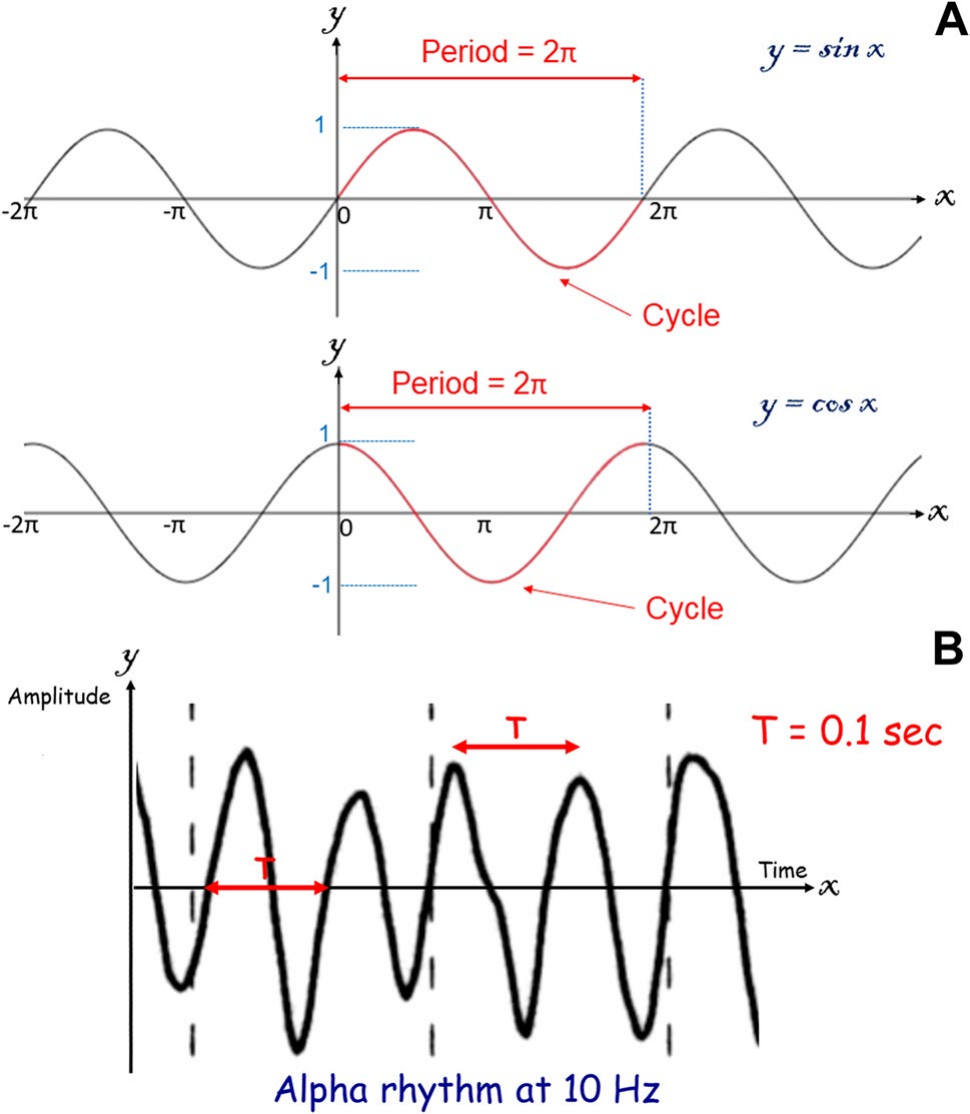
**A** Sine and cosine functions are called periodic functions because the pattern continues indefinitely. The time interval containing one copy of the repeating pattern is called the period. **B** Example of a periodic function according to mathematicians with an alpha rhythm at 10 Hz. The period (T) is 0.1 s (1/10)

**Fig. 3:**
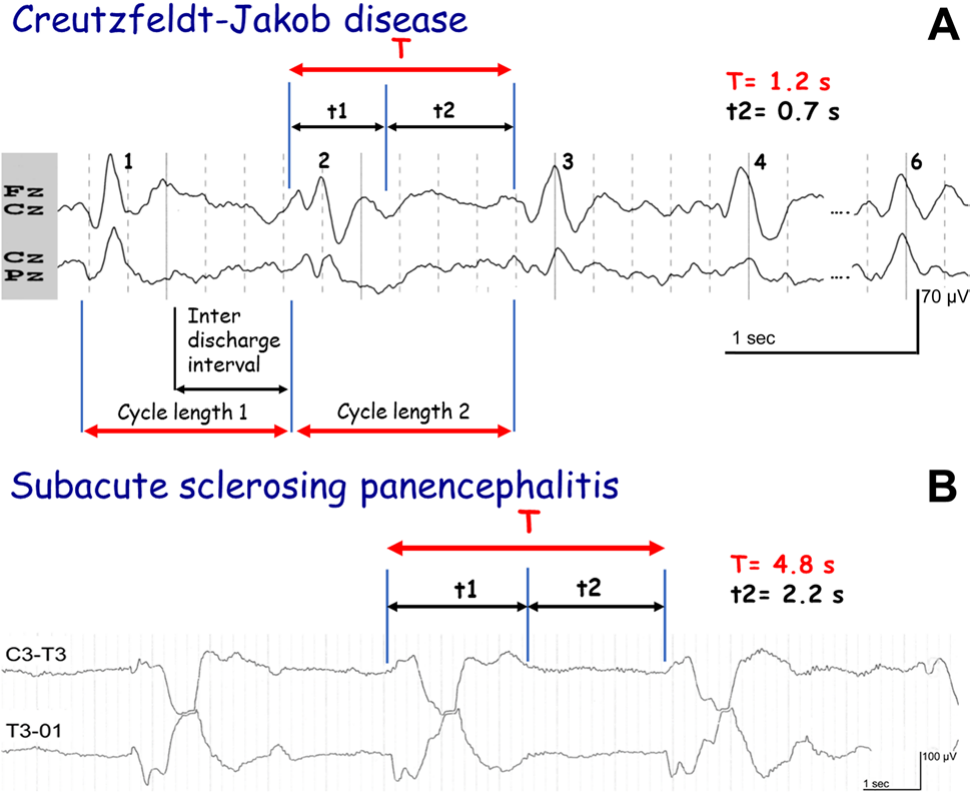
**A** 88-year-old man with Creutzfeldt–Jakob disease. Periodic complexes at 1–1.5 Hz that allow us to classify this pattern among bilateral synchronous short-period activities according to Gaches (0.5–4 s) [4] and periodic short-interval diffuse discharges according to Brenner and Schaul (0.5–4 s) [13]. **B **Subacute sclerosing panencephalitis in a 15-year-old man not vaccinated against measles. Severe cognitive decline was observed. Periodic high amplitude complexes every 4.5 s corresponding to Radermecker complexes. The complexes have a “squared” aspect due to signal saturation (non-digital recording). Please be aware of the discrepancy between Gaches and Brenner and Schaul’s classifications. According to Gaches, this EEG corresponds to a bilateral synchronous long-period pattern (*T* > 4 s), however, according to Brenner and Schaul, there are periodic short-interval diffuse discharges (t2 < 4 s)

**Fig. 4:**
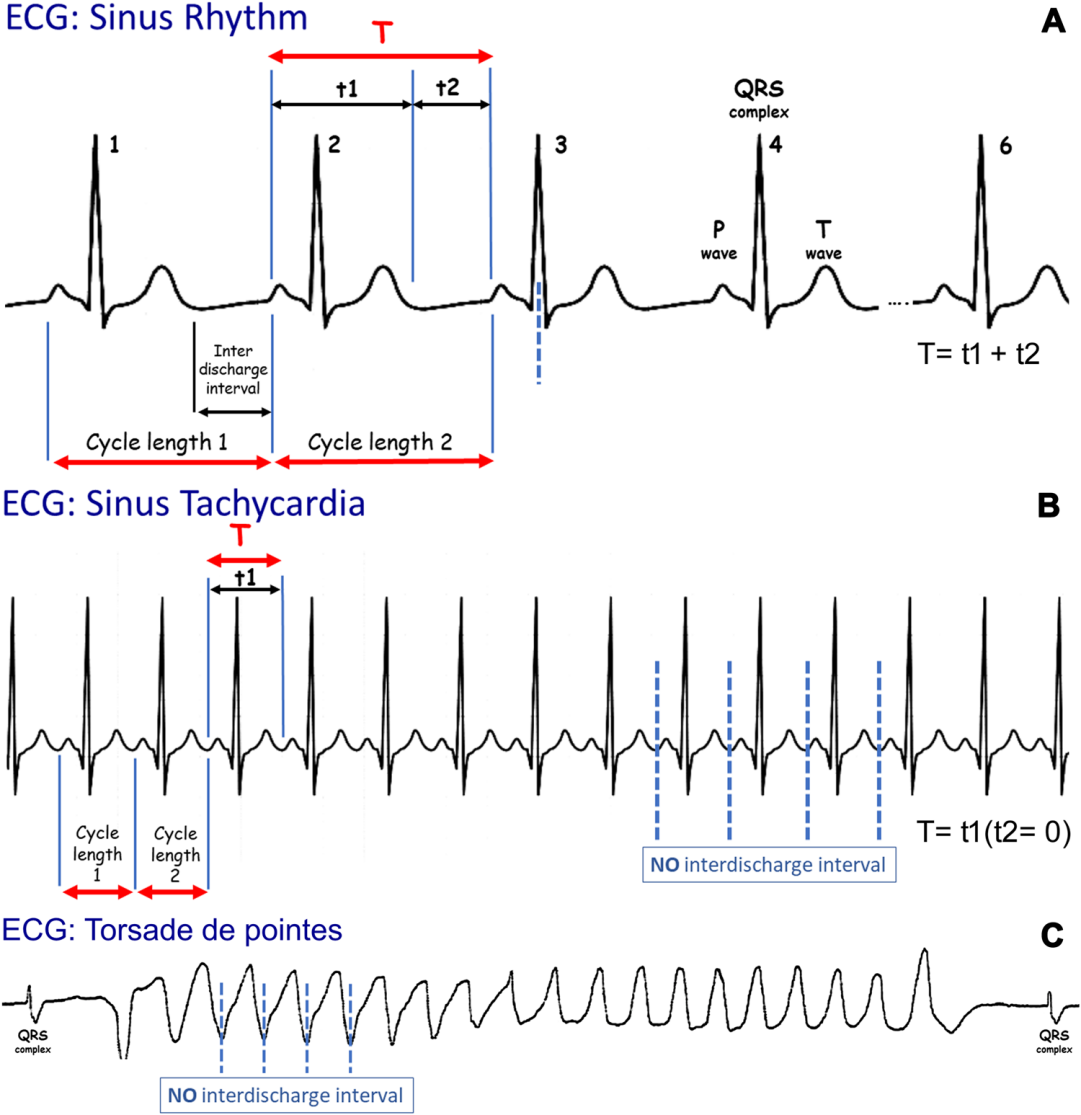
Comparison between sinus rhythm (**A**) and sinus tachycardia (**B**). In this situation, there is no apparent inter-discharge interval (t2 = 0) but QRS complexes occur at regular time intervals. The ECG and the motion of the heart are periodic. **C** Torsade de pointes episode triggered by a premature ventricular contraction on the T wave in a patient with a long QT interval. Torsade de pointes is a type of ventricular tachycardia. Make a comparison between figure B (periodic pattern with no inter-discharge interval) and this figure (rhythmic pattern for neurophysiologists) which corresponds to a cardiac rhythm disorder

In mathematics, a graph which consists of a repeated pattern over a constant interval is described as periodic [2], each pattern in the graph being called a cycle. The time interval containing one copy of the repeating pattern is called the period, T (Fig. 2) [3]. T is the sum of the duration of the wave (t1) and the duration of the interval between two consecutive waves (t2): *T* = t1 + t2 (Figs. 3, 4). In electroencephalography, the notion of periodic patterns was introduced after the second world war with the notion of stereotyped paroxysmal complexes occurring at regular or nearly regular intervals, i.e., the period (*T*) [4]. According to the American Clinical Neurophysiology Society (ACNS) standardized critical care EEG nomenclature (2021), periodic EEG discharges correspond to “the repetition of waveforms with relatively uniform morphology and duration with a clearly discernible inter-discharge interval between consecutive waveforms and recurrence of the waveform at nearly regular interval” [5]. The only difference between periodic and rhythmic EEG patterns is the break between each discharge. Spike-and-wave or sharp-and-wave also represent specific EEG patterns with no break between discharges [5]. They consist of alternating patterns of spike/sharp-wave followed by a slow wave.

ACNS includes in the definition of periodic EEG patterns the requirement of an inter-discharge interval [5]. However, some periodic patterns exist without a substantial inter-discharge interval or one that is minimally present or absent. Combined terminology has been included in the ACNS terminology of EEG in critically ill patients such as lateralized periodic discharges (LPDs) with superimposed rhythmic delta activity. When triphasic waves (TWs) are encountered, they are no longer identified as generalized periodic discharges (GPDs) with triphasic morphology when an inter-discharge interval does not exist and instead should now be considered as complexes of continuous, diffuse, sharp-and-slow waves. The aim of this paper is to promote a mathematical approach to waveforms in the standard EEG, based upon historical development of the concept of what defines periodic EEG activity. We suggest alternative definitions be considered beyond the current use of "*periodic*" and "*rhythmic*" as terminology used when interpreting critical care EEGs. By advancing constructs based on pre-established mathematical concepts, terminology will achieve greater specificity and consistency. Enhanced understanding of terminology will underscore mechanisms of seizure urgencies and emergencies and help avoid overinterpretation of an ictal pattern as rhythmic along the ictal–interictal continuum (IIC).

## Periodic EEG patterns

The concept of periodicity was first introduced to describe Pette–Döring encephalitis during the second world war [4]. This was most likely, a term used to describe subacute sclerosing panencephalitis (SSPE). This concept of periodicity was formally used a few years later by Radermecker (1949) with the description of periodic complexes seen in SSPE [6], which in turn, led to the delineation of this entity [7]. Bickford and Butt (1955) described TWs in hepatic encephalopathy [8], an EEG pattern previously referred to as “*blunted spike-and-wave*” [9]. The concept of periodicity was then applied to Creutzfeldt–Jakob disease (CJD) [10], post-anoxic syndrome [4, 11], and repetitive focal or lateralized sharp wave discharges [12].

One of the first comprehensive approaches to this subject was carried out by Gaches, who classified periodic EEG activities according to their distribution (focal/lateralized versus bilateral synchronous) and especially according to their periodicity (between 0.5 and 4 s, i.e., a short period versus between 4 and 30 s, i.e., long period) to potentially represent an etiology before present day sophistication of neuroimaging procedures [4]. Brenner and Schaul used a classification system similar to the one used by Gaches and classified periodic EEG patterns according to the topographic distribution of the complexes, however, they considered within their classification the interval between discharges, and not the period. They defined a short interval as varying between 0.5 and 4 s and a long interval as varying between 4 and 30 s. Based on these features, they classified periodic EEG patterns into LPDs, bilateral independent periodic discharges, periodic short-interval diffuse discharges, and periodic long-interval diffuse discharges [13]. These were the same time limits which were proposed by Gaches, i.e., between 0.5 and 4 s and > 4 s, but are not applied at the same interval. For a specific pathology (CJD, SSPE…), the assessment of the period is reproducible, but not the interval between discharges (t2), because the duration of the complex (t1) may differ. Consequently, a different conclusion can be reached for the same EEG pattern (Fig. 3B).

## Historical definitions

Gaches proposed defining periodic activities as “stereotyped repetitive activities of prolonged expression”. Stereotyped because the complexes have a constant morphology throughout the tracing, repetitive due to the occurrence of the same complex with a constant or nearly constant interval, and prolonged expression because periodic phenomena must be observed over a sufficient length of time [4]. Along the same line of reasoning, Kuroiwa and Celesia defined periodic patterns “as stereotyped paroxysmal complexes separated by nearly identical intervals” [14]. In their article, they cite an example of “generalized periodic triphasic waves in a 60-year-old man in hepatic coma” without including a clearly identifiable inter-discharge interval between consecutive waveforms. According to Shear, “periodic EEG activity is EEG activity that occurs, appears, or recurs at regular intervals from time to time or intermittently” [15].

The definition of the International Federation of Clinical Neurophysiology for the term periodic mentions the occurrence of waveforms or complexes at an approximately regular rate or at approximately regular intervals, generally of one to several seconds [16, 17]. This definition is still valid [18]. Following early recommendations for terminology and classification of critical care EEG by the ACNS [19], a specific definition for periodic discharges was added to address the quantifiable inter-discharge interval between relatively uniform consecutive waveforms that occur at regular intervals [18].

## Periodic function

From a mathematical point of view, the historical definitions of a periodic EEG with stereotyped complexes at constant or nearly constant intervals are exact. A phenomenon is periodic when it is morphologically the same and recurs at regular time intervals (Fig. 2). The duration between two successive repetitions is called the period but should not be confused with the inter-discharge interval, i.e., t2 (Figs. 3, 4). The periodicity is defined by the time T sum of the duration of the complex (t1) and the duration of the interval (t2), which separates it from the following complex. The mathematical period (T) is termed cycle length in ACNS definition [5] (Figs. 3, 4).

EEG interpretation can be compared to ECG interpretation: P-QRS-T complexes correspond to t1, the interval *T* to *P* corresponds to t2, and the P-to-P interval corresponds to *T* (Fig. 4A). At rest, t2 is clearly discernible, but after physical effort, when pulse rates are more than 100 beats per minute, this interval can disappear (Fig. 4B). Even if physical activity can modify the rhythm and influence t2 (no inter-discharge interval), normal heart rate represents a typical physiological periodic phenomenon. In atrial fibrillation, QRS complexes occur at irregular time intervals; therefore, heartbeats are not periodic. In ventricular tachycardia, there is no inter-discharge interval between the delta waves and the ECG pattern could be described by neurophysiologists as rhythmic delta waves, i.e., not periodic (Fig. 4C). Cardiologists refer this clinical phenomenon as arrhythmia because the term arrhythmia refers to abnormal electrical activity in the heart [20].

With a truly mathematical definition, most EEG patterns are periodic, except when the eyes are open (desynchronization) because there are no repetitive phenomena unless lambda waves occur, in the case of abnormal continuous polymorphic delta slowing, or evolving waveforms during seizures. Typically, seizures in patients with epilepsy have low-voltage frequencies at seizure onset that increase in amplitude and decrease in frequency with propagation to adjacent regions of the brain. As a result, changes in amplitude and frequency during seizure evolution are not considered periodic. However, there are exceptions with relative fluctuation (e.g., LPDs, especially LPDs-max), that may reflect non-convulsive status epilepticus [21]. For mathematicians, trigonometric/sinusoidal functions are periodic (Fig. 2A). Therefore, the alpha rhythm at 10 Hz is a periodic function with a period between two waves of 0.1 s (*T* = 1/10) (Fig. 2B). Three-Hz generalized spike-wave discharges of childhood absence epilepsy have a period of 0.33 s between the spikes (*T* = 1/3).

## Recent evolution of the concept

The first definitions of periodic EEG included the occurrence of waveforms or complexes recurring at nearly regular intervals (i.e., T, the period) [4, 14, 15, 17]. In 2005, the ACNS adopted a change in the definition, to consider the t2 component [22]. Since then, the concept of periodic discharges has evolved from “a quantifiable inter-discharge interval between consecutive waveforms” [19] to “a clearly discernible inter-discharge interval,” [5]. TWs are periodic for EEGers, and the ACNS proposed changing the name TWs to “GPDs with triphasic morphology”. The goal was to eliminate terms with an implied clinical connotation.

**Fig. 5:**
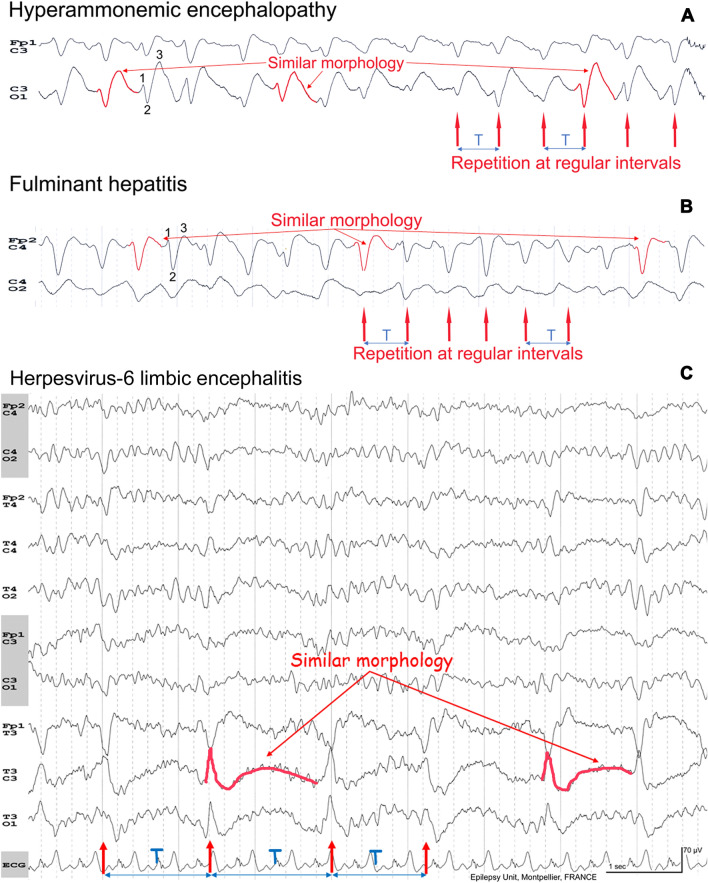
**A**, **B** Nearly continuous triphasic waves (TWs) with no apparent inter-discharge interval (t2 = 0). These are repetitive and stereotyped paroxysmal complexes occurring at a nearly constant interval allowing us to classify these activities as periodic TWs and not sharp-and-waves. **C** Lateralized periodic discharges (LPDs) over the left temporal region with no apparent inter-discharge interval between the spike-and-wave complexes. It is more important to recognize the repetitive and the stereotypic nature of the pattern rather than describe this pattern as LPDs with superimposed rhythmic delta activity because there is no inter-discharge interval

When TWs appear nearly continuously, the intervals between the complexes are so short that they may appear absent with no apparent background between the complexes (Fig. 5A, B). In toxic–metabolic encephalopathies, as with other etiologies of encephalopathy, TWs may occur with the first clinical symptoms singly, then with the progression of the disease in short runs, in clusters, and then continuously (Fig. 1B). This can give the false impression of slow spike-waves or sharp-and-waves. Before appearing as a continuous pattern, TWs may variably mix with slow waves [8]. TWs are also influenced by the level of vigilance (fully awake versus drowsy versus asleep) [23, 24]. Despite these fluctuations, reading these EEGs is like watching a boring movie. The waves can fluctuate, but the EEG appears at the end monotonous/monomorphic.

Using the terminology of generalized sharp-and-wave activity, because there is no apparent inter-discharge interval instead of generalized periodic TWs represents a leap backwards over 70 years. From a historical standpoint, one needs to remember that TWs were initially described as "blunted spike and wave" [9], and then later named TWs by Bickford and Butt (1955) who included in their paper a typical example of TWs with a mix of slow waves and no apparent interval between the waves in a patient with “semicoma” due to hepatic encephalopathy [8].

The word rhythmic in the ACNS terminology applies to delta waves and means “repetition of a waveform with relatively uniform morphology and duration and without an interval between consecutive waveforms” [5]. Rhythmic delta waves may be arciform and sinusoidal but may also be complex [5]. TWs are by definition complex waveforms typically comprised of delta waves with three phases (Figs. 5A, B, 7). When they occur nearly continuously without an apparent inter-discharge interval, they should be defined as rhythmic complex delta waves instead of periodic, with the risk of interpreting this pattern as ictal or on the IIC. However, TWs are now identified as “GPDs with triphasic morphology”. Given the frequency, there is no need to identify the intervals between the complexes. Only the morphology with the three phases of a TW can be used to qualify the pattern as periodic. More important than the interval between two consecutive complexes, TWs are stereotypic and repetitive paroxysmal complexes (Figs. 5A, B, 7). Thus, it seems more important to recognize the repetitive and stereotypic nature of the pattern with the three phases than an inter-discharge interval.

The concept of clearly discernible inter-discharge intervals is usually easy to recognize for LPDs, but sometimes, there is no apparent inter-discharge interval when the activity consists of focal spike- or sharp-wave complexes (Fig. 5C). Similarly, it is more important to recognize the repetitive and the stereotypic nature of the pattern rather than describe this pattern as LPDs with superimposed rhythmic delta activity, because there is no inter-discharge interval [5].

## Proposed definition

**Fig. 6:**
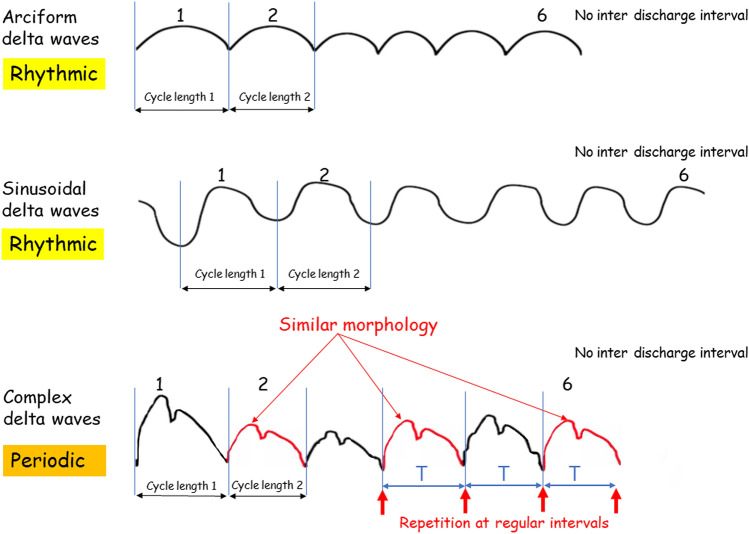
Comparison between arciform/sinusoidal delta waves and complex delta waves at 2 or below 2 Hz. There is a basic morphology for rhythmic EEG patterns and a complex morphology for periodic EEG patterns; these are repetitive and stereotyped paroxysmal complexes occurring at a constant interval, allowing us to classify this pattern as periodic even if there is no discernible inter-discharge interval. For periodic EEG patterns, the regularity of the time interval (T) supersedes the inter-discharge interval (t2)

**Fig. 7:**
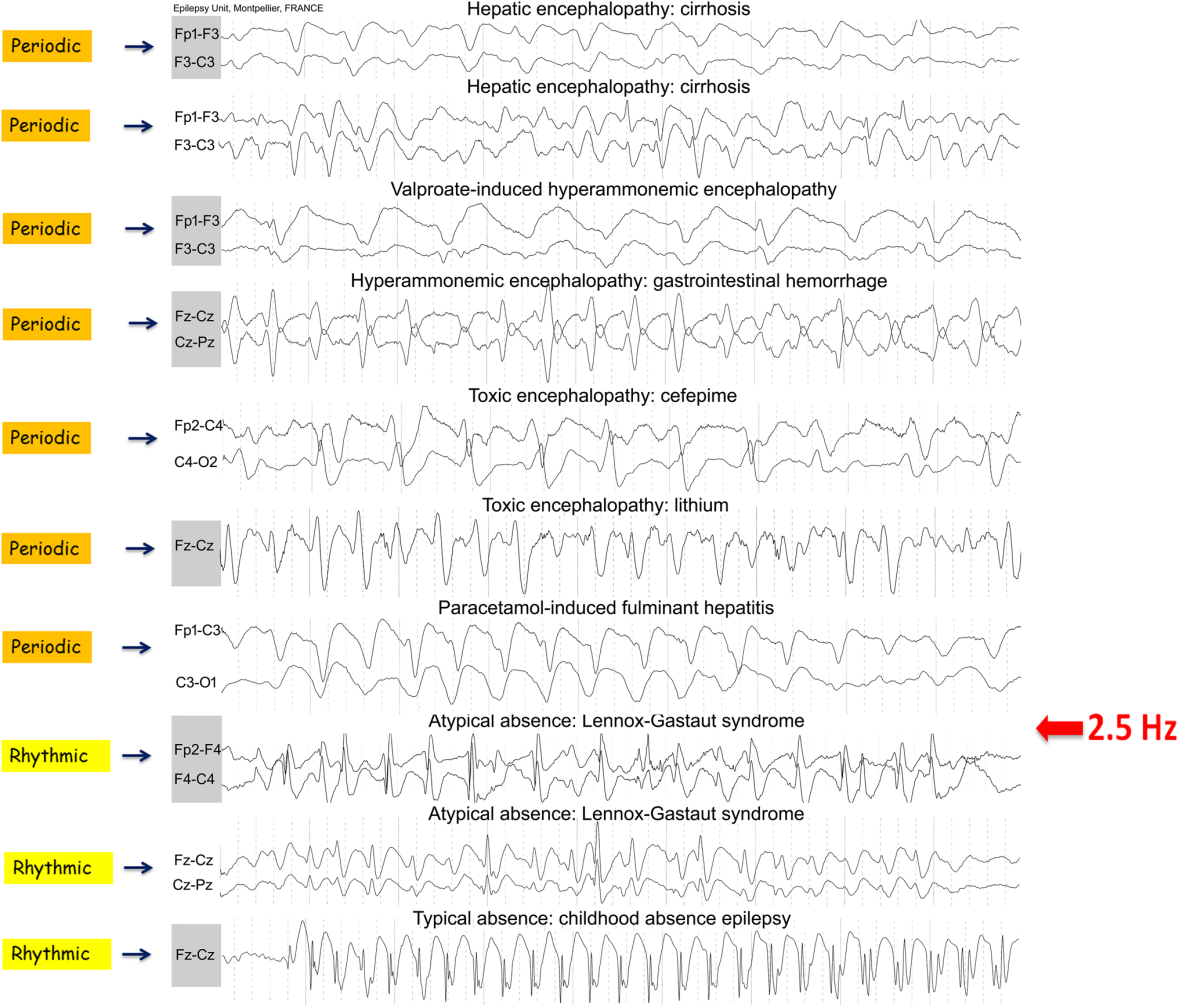
Comparison among triphasic waves (TWs) with no apparent inter-discharge interval and rhythmic spike-waves. The reversal point between periodic TWs and spike-waves is between 2 and 2.5 Hz. Indeed, by definition, slow spike-and-waves at 2.5 Hz in Lennox–Gastaut syndrome for EEGers are rhythmic and TW at 2 or less than 2 Hz are periodic. The American Clinical Neurophysiology Society proposed changing the name TWs to “continuous 2/s Generalized Periodic Discharges with triphasic morphology” [19]

Paroxysmal EEG activity stands out clearly from the background rhythm and must be repetitive and stereotypic to qualify this pattern as periodic. Periodic patterns usually consist of spikes, sharp waves, polyspikes, complex delta waves, and TWs. When spikes or sharp waves occur on an isoelectric background (typically in anoxia), it is easy to spot the inter-discharge interval and qualify the pattern as periodic, whatever the number of cycles per second involved. But, for complex delta waves that occur continuously or nearly continuously, it is essential to establish a cutoff value in terms of frequency to differentiate periodic versus rhythmic EEG patterns. For example, terminology identifying slow spike-waves in Lennox–Gastaut syndrome that recur at 2.5 Hz are referred to as rhythmic, while TWs at 2 Hz are considered periodic (continuous 2/s GPDs with triphasic morphology [19]). The distinction between rhythmic and periodic for complex delta waves, therefore, lies somewhere between 2 and 2.5 Hz (Fig. 7). While TWs at 2 Hz are considered periodic, sinusoidal or arciform delta waves at 2 Hz or below are rhythmic. It is important to consider that the detailed morphology of delta waves should also be considered in periodic patterns that involve a complex morphology (e.g., the three phases for TWs) vs a simple morphology involving rhythmic patterns in the EEG at 2 Hz or less (Fig. 6).

How long must the repetitive phenomenon be recorded to identify the activity as periodic? Gaches (1971) proposed 30 min [4], Dunand and Jallon (2002) at least 20 min [25] corresponding to the duration of a standard EEG recording. Kuroiwa and Celesia (1980) felt periodic patterns had to be present in the entire tracing or continuously for at least a 10-min epoch, and if they occurred for epochs of less than 10 min, they had to be continuous during a specific behavioral state [14]. To align with the ACNS criteria, a pattern is able to be identified as rhythmic or periodic if and only if it continues for at least six cycles (e.g., 1 Hz for 6 s, or 3 Hz for 2 s) [5] (Fig. 3, 4). Often, ictal EEGs in patients with epilepsy terminate with paroxysmal intervals that gradually wane in frequency over time. Therefore, a 10–20-s epoch analyzing only the terminus of the seizure could qualify as periodic despite the entire EEG recording not being representative of a pattern that is stereotyped.

No well-defined limit to pattern duration is universal. The interpretation of EEG duration, therefore, depends on the pattern morphology, periodicity (short versus long period), and the experience of the EEG interpreter. It is easy to recognize the periodic character when TWs occur nearly continuously in a 10–20-s epoch because the morphology is characteristic and the pattern is monomorphic. However, in other situations, it is necessary to view the entire EEG to determine the monotonous/monomorphic aspect of the complexes. Overall, the duration of the recording must be long enough to prove that the pattern can be qualified as periodic when it is repetitive and stereotyped.

We propose returning to the initial definitions used historically to describe EEG patterns. To do so requires a uniform approach refocusing on the period (*T*) when using keywords such as “stereotyped, repetitive and prolonged expression” as proposed by Gaches [4] and “separated by nearly identical intervals” proposed by Kuroiwa and Celesia [14]. Periodic patterns were previously defined in the EEG as “any runs of stereotyped paroxysmal waveforms separated by nearly identical intervals and prolonged repetitive complexes on the EEG”. Prolonged expression referred to EEGs recorded for a sufficient time to prove that the pattern has a uniform morphology and occurs at constant or nearly constant intervals, resulting in a monomorphic and monotonous EEG recording.

## Conclusion

EEG terminology was previously developed to incorporate etiology prior to present-day sophisticated neuroimaging availability. We suggest that periodic EEG features should be considered in the context of past descriptions of EEG patterns. No inter-discharge interval is necessary when describing periodic EEG patterns as stereotyped and occurring at regular and similar intervals. For periodic EEG patterns, the regularity of the time interval (T) supersedes the inter-discharge interval (t2). As a result, periodic EEG activity should be considered along a continuum, not in direct opposition to rhythmic EEG activity where no interval activity exists between consecutive waveforms (Fig. 7). When recurring delta waveforms are present, and morphology is complex, failure to recognize the periodic pattern may lead to misdiagnosis of the EEG as rhythmic and lead to over-interpretation as an ictal pattern lying along the IIC. We support universal use of terminology in EEG interpretation to limit the potential for misdiagnosis and optimize treatment for patients with potential seizure urgencies and emergencies.

## Data Availability

No data are associated with this article.

## References

[1] Tatum WO, Olga S, Ochoa JG, Munger Clary H, Cheek J, Drislane F (2016). American Clinical Neurophysiology Society Guideline 7: guidelines for EEG reporting. J Clin Neurophysiol.

[2] Clarke D. Heinemann Higher Mathematics*.* Pearson Education; 1998.

[3] Pemberton S (2018). Cambridge IGCSE® and O Level Additional Mathematics Coursebook.

[4] Gaches J (1971). Activités périodiques en E.E.G [Periodic activity in the EEG]. Rev Electroencephalogr Neurophysiol Clin.

[5] Hirsch LJ, Fong MWK, Leitinger M, LaRoche SM, Beniczky S, Abend NS (2021). American Clinical Neurophysiology Society's Standardized Critical Care EEG Terminology: 2021 Version. J Clin Neurophysiol.

[6] Radermecker J (1949). Aspects électroencéphalographiques dans trois cas d'encéphalite subaigue. Acta Neurol Psychiatr Belg.

[7] Cobb W, Hill D (1950). Electroencephalogram in subacute progressive encephalitis. Brain.

[8] Bickford RG, Butt HR (1955). Hepatic coma: the electroencephalographic pattern. J Clin Invest.

[9] Foley JM, Watson CW, Adams RD (1950). Significance of the electroencephalographic changes in hepatic coma. Trans Am Neurol Assoc.

[10] Jones DP, Nevin S (1954). Rapidly progressive cerebral degeneration (subacute vascular encephalopathy) with mental disorder, focal disturbances, and myoclonic epilepsy. J Neurol Neurosurg Psychiatry.

[11] Spoerel WE (1962). The electroencephalogram after cardiac arrest. Can Anaesth Soc J.

[12] Chatrian GE, Shaw CM, Leffman H (1964). The significance of periodic lateralized epileptiform discharges in EEG: an electrographic, clinical and pathological study. Electroencephalogr Clin Neurophysiol.

[13] Brenner RP, Schaul N (1990). Periodic EEG patterns: classification, clinical correlation, and pathophysiology. J Clin Neurophysiol.

[14] Kuroiwa Y, Celesia GG (1980). Clinical significance of periodic EEG patterns. Arch Neurol.

[15] Schear HE (1984). Periodic EEG activity. Clin Electroencephalogr.

[16] Noachtar S, Binnie C, Ebersole J, Mauguière F, Sakamoto A, Westmoreland B (1999). A glossary of terms most commonly used by clinical electroencephalographers and proposal for the report form for the EEG findings. The International Federation of Clinical Neurophysiology. Electroencephalogr Clin Neurophysiol Suppl.

[17] Chatrian GE (1975) In: The VIIIth International Congress of Electroencephalography and Clinical Neurophysiology Proceedings of the General Assembly. Am J EEG Technol 15(4):171–200. 10.1080/00029238.1975.11081045

[18] Kane N, Acharya J, Beniczky S, Caboclo L, Finnigan S, Kaplan PW (2017). A revised glossary of terms most commonly used by clinical electroencephalographers and updated proposal for the report format of the EEG findings. Revision 2017. Clin Neurophysiol Pract.

[19] Hirsch LJ, LaRoche SM, Gaspard N, Gerard E, Svoronos A, Herman ST (2013). American Clinical Neurophysiology Society's Standardized Critical Care EEG Terminology: 2012 version. J Clin Neurophysiol.

[20] World Health Organization. ICD-11. International Classification of Diseases 11th Revision; https://icd.who.int/en.

[21] Gelisse P, Crespel A, Genton P, Jallon P, Kaplan PW (2021). Lateralized periodic discharges: which patterns are interictal, ictal, or peri-ictal?. Clin Neurophysiol.

[22] Hirsch LJ, Brenner RP, Drislane FW, So E, Kaplan PW, Jordan KG (2005). The ACNS subcommittee on research terminology for continuous EEG monitoring: proposed standardized terminology for rhythmic and periodic EEG patterns encountered in critically ill patients. J Clin Neurophysiol.

[23] Gelisse P, Crespel A, Gigli GL, Kaplan PW (2021). Stimulus-Induced Rhythmic or Periodic Intermittent Discharges (SIRPIDs) in patients with triphasic waves and Creutzfeldt-Jakob disease. Clin Neurophysiol.

[24] Gelisse P, Crespel A, Thomas P, Jallon P, Genton P, Kaplan PW (2021). Is Socrates a cat? False EEG syllogisms in critically ill patients. Clin Neurophysiol.

[25] Dunand A-C, Jallon P (2002). Les activités paroxystiques pseudo-périodiques en électroencéphalographie [Pseudoperiodic and paroxysmal electroencephalographic activities]. Neurophysiol Clin.

